# Supplementary material for: Short- and Long-Interval Prime-Boost Vaccination with the Candidate Vaccines MVA-SARS-2-ST and MVA-SARS-2-S Induces Comparable Humoral and Cell-Mediated Immunity in Mice
Source: Viruses. 2023 May 17;15(5):1180. doi: 10.3390/v15051180 (PMC10222707; doi:10.3390/v15051180)
Supplement: Supplementary file 1 [file viruses-15-01180-s001.zip › viruses-2401380-supplementary.pdf]

Supplementary Table S1. Recombinant SARS-CoV-2 S proteins used for ELISA.

Recombinant Protein	Company	Catalogue No.
SARS-CoV-2 S protein, His Tag	ACROBiosystems	SPN-C52H4
SARS-CoV-2 (COVID-19) S1 protein, His Tag	ACROBiosystems	S1N-C52H3
SARS-CoV-2 Spike Glycoprotein (S2), Sheep Fc-Tag	The Native Antigen Company	REC31807-500
SARS-CoV-2 (COVID-19) S protein RBD, His Tag	ACROBiosystems	SPD-C52H3

Supplementary Table S2. Antibodies and staining reagents for flow cytometric analysis of mouse splenocytes.

Antigen	Clone	Fluorophore	Company	Catalogue No.
CD3	17A2	PE-Cy7	Biolegend	503832
CD4	GK1.5	Brilliant Violet 421	Biolegend	100437
CD8α	53-6.8	Alexa Fluor 488	Biolegend	100723
CD16/CD32	93	N/A	Biolegend	101320
IFN-γ	XMG1.2	APC	Biolegend	505810
TNF-α	MP6-XT22	PE	Biolegend	506306
Viability	N/A	Zombie Aqua	Biolegend	423101

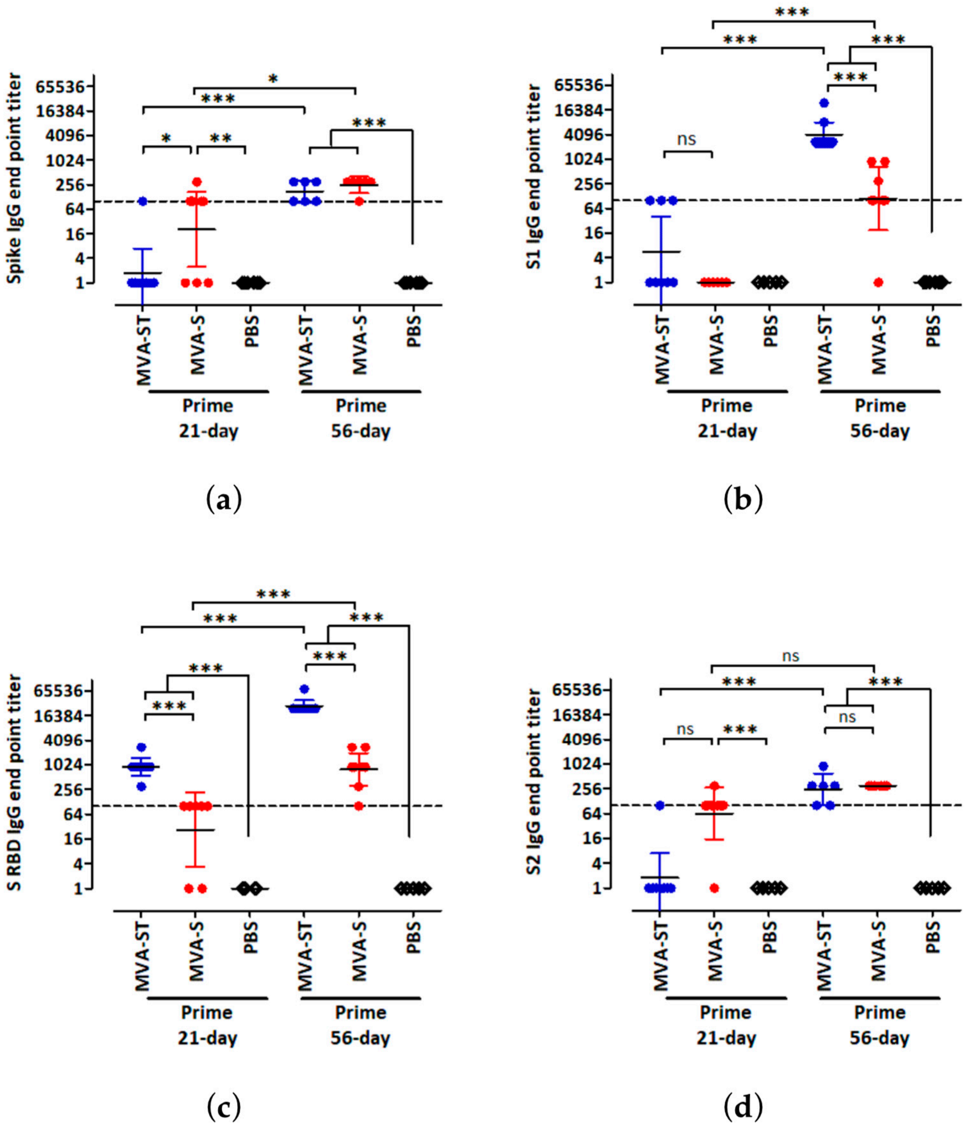

Supplementary Figure S1. Spike antigen-specific antibodies induced after prime immunization with MVA-SARS-2-ST and MVA-SARS-2-S. Groups of BALB/c mice (n = 6 to 8) were immunized twice over a 21-day or a 56-day interval with 10^8 PFU of MVA-SARS-2-ST (MVA-ST) and MVA-SARS-2-S (MVA-S) via the intramuscular (i.m.) route. Mice inoculated with saline (PBS) served as controls. Sera were collected 18 days (21-day schedule) or 53 days (56-day schedule) after the first immunization (prime) and analyzed for SARS-2 full length spike (**a**), S1 (**b**), receptor binding domain (RBD) (**c**) and S2 (**d**) specific IgG binding titers by ELISA. Graphs show normalized data after prime immunization and dashed lines represent the limits of detection (LOD). Bars represent the geometric mean + 95% CI. For statistical analysis log transformed data were analyzed by one-way ANOVA and Tukey post test. Asterisks represent statistically significant differences between two groups. *** $p < 0.001$, ** $p < 0.01$, * $p < 0.05$, ns = not significant

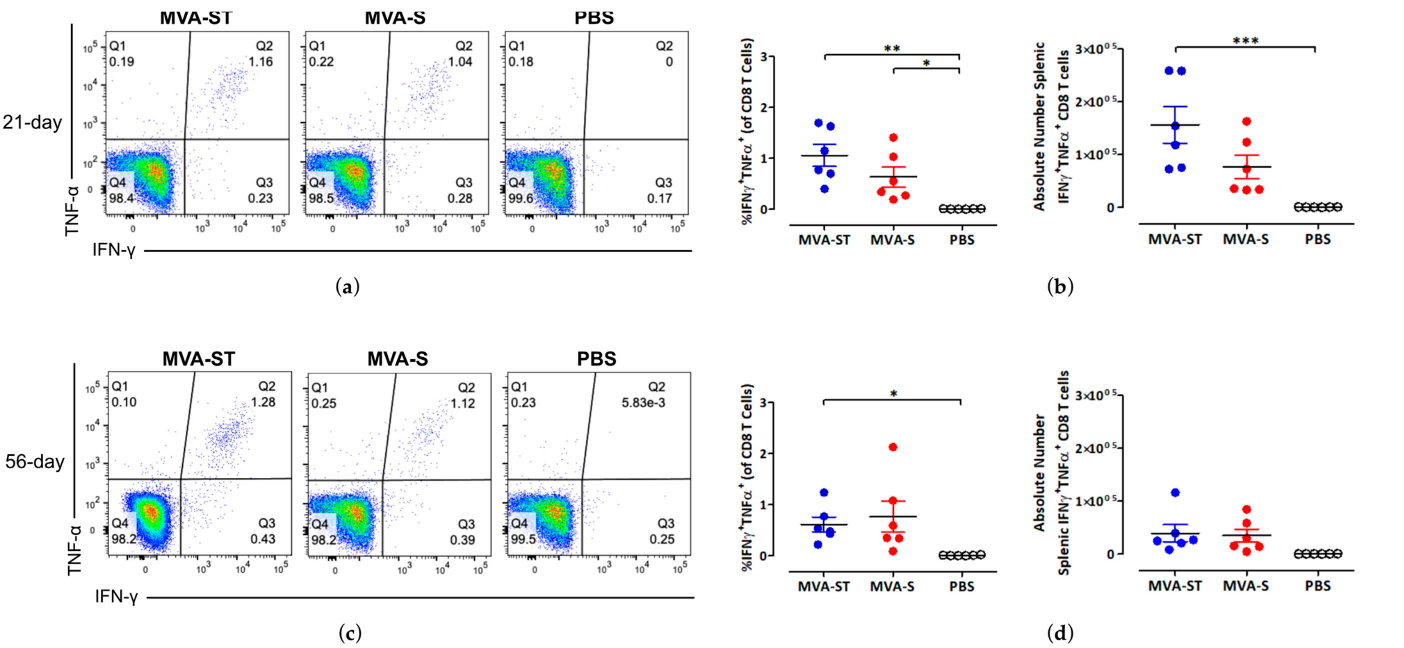

Supplementary Figure S2. IFN-γ and TNF-α production by activated SARS-2 S-specific CD8 T cells after prime-boost immunization (21-day and 56-day intervals) with MVA-SARS-2-ST and MVA-SARS-2-S. Groups of BALB/c mice (n = 6) were immunized twice with 10^8 PFU MVA-ST or MVA-S over a 21-day (**a**,**b**) or a 56-day interval (**c**,**d**) via the i.m. route. Saline immunized mice (PBS) served as negative control. Splenocytes were collected and prepared on day 14 after the booster immunization. Total splenocytes were stimulated with the H2-d restricted peptide of the SARS-2-S protein $S_{268-276}$ (GYLQPRTFL) and were measured by IFN-γ and TNF-α ICS plus FACS analysis. (**a**,**c**) Representative flow cytometry plots showing IFN-γ and TNF-α production in the CD8 T cells compartment after stimulation. (**b**,**d**) Graphs showing frequency and absolute number of IFN-γ+TNF-α+ cells within the CD8 T cell compartment. Bars represent the mean + SEM. Differences between groups were analyzed by one-way ANOVA and Tukey post test. Asterisks represent statistically significant differences between two groups. *** $p < 0.001$, ** $p < 0.01$, * $p < 0.05$

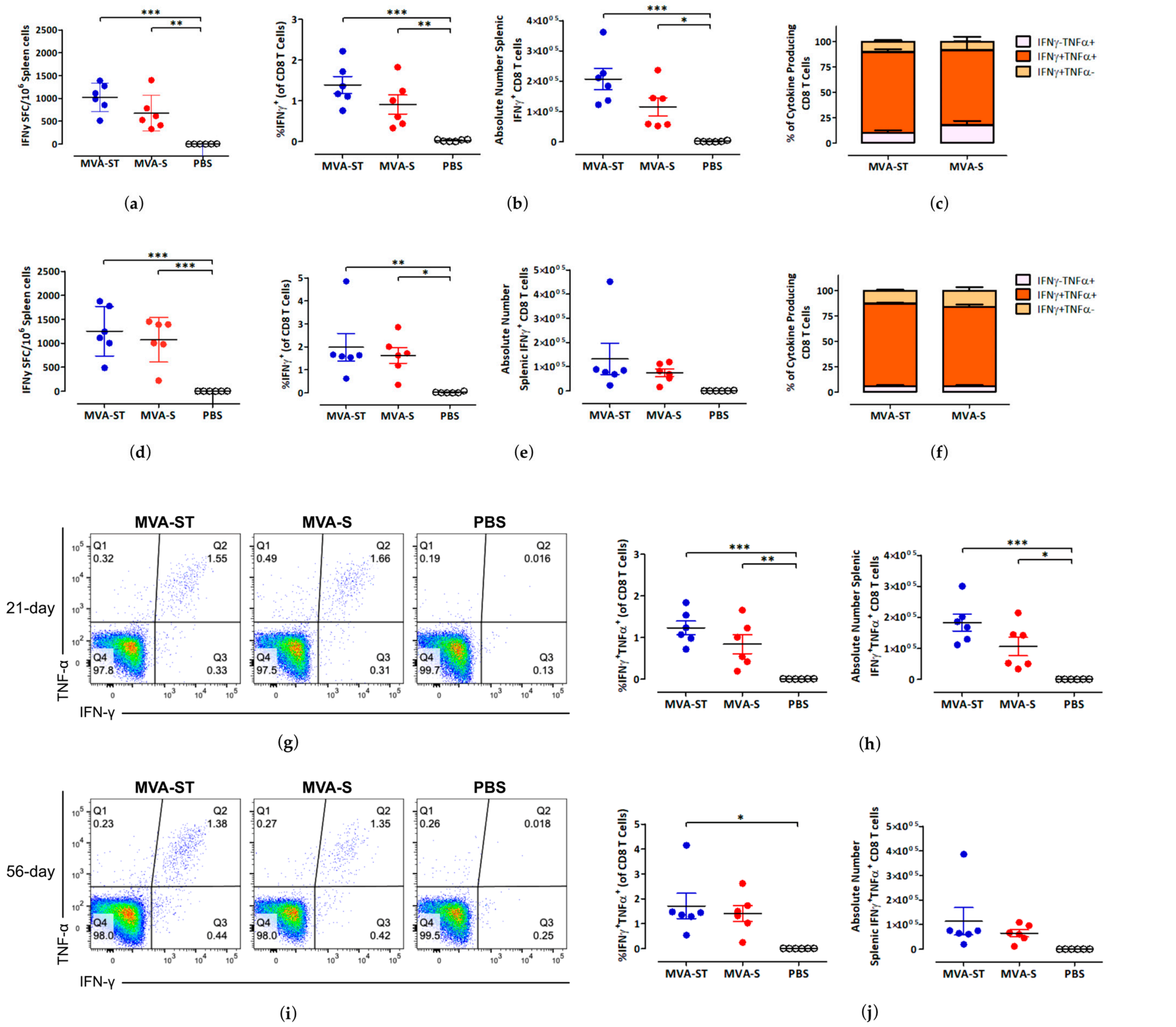

Supplementary Figure S3. Activation of MVA-specific CD8 T cells after prime-boost immunization (21-day and 56-day intervals) with MVA-SARS-2-ST and MVA-SARS-2-S. Groups of BALB/c mice (n = 6) were vaccinated twice with 10^8 PFU of MVA-SARS-2-ST (MVA-ST) and MVA-SARS-2-S (MVA-S) over 21-day interval (**a-c** & **g-h**) or 56-day interval (**d-f** & **j-i**) via the i.m. route. Saline immunized mice (PBS) served as control. Splenocytes were collected and prepared 14 days after the booster immunization. Total splenocytes were stimulated with the H2d restricted MVA-specific peptide $F2_{26\text{-}34}$ and measured by IFN-γ ELISPOT assay and IFN-γ and TNF-α ICS plus FACS analysis. (**a,d**) IFN-γ spot forming colonies (SFC) for stimulated splenocytes measured by ELISPOT assay. (**b,e**) IFN-γ production by CD8 T cells measured by FACS analysis. Graphs show the frequency and absolute number of IFN-γ+ CD8 T cells. (**c,f**) Cytokine profile of $F2_{26\text{-}34}$ -specific CD8 T cells. Graphs show the mean frequency of IFN-γ-TNF-α+, IFN-γ+TNF-α+ and IFN-γ+TNF-α- cells within the cytokine positive CD8 T cell compartment. Bars represent the mean + SEM. Differences between groups were analyzed by one-way ANOVA and Tukey post test. Asterisks represent statistically significant differences between two groups. (**g,i**) Representative flow cytometry plots showing IFN-γ and TNF-a production in the CD8 T cells compartment after stimulation. (**h,j**) Graphs showing frequency and absolute number of IFN-γ +TNF-α+ cells within the CD8 T cell compartment. Bars represent the mean + SEM. Differences between groups were analyzed by one-way ANOVA and Tukey post test. Asterisks represent statistically significant differences between two groups. *** $p < 0.001$, ** $p < 0.01$, * $p < 0.05$
